# Finding food in a novel environment: The diet of a reintroduced endangered meso-predator to mainland Australia, with notes on foraging behaviour

**DOI:** 10.1371/journal.pone.0243937

**Published:** 2020-12-17

**Authors:** Natasha M. Robinson, Wade Blanchard, Christopher MacGregor, Rob Brewster, Nick Dexter, David B. Lindenmayer

**Affiliations:** 1 Fenner School of Environment and Society, The Australian National University, Canberra, Australian Capital Territory, Australia; 2 National Environmental Science Program, Threatened Species Recovery Hub, Fenner School of Environment and Society, The Australian National University, Canberra, Australian Capital Territory, Australia; 3 Rewilding Australia, Sydney, New South Wales, Australia; 4 Booderee National Park, Jervis Bay, Jervis Bay Territory, Australia; Universidad Austral de Chile, CHILE

## Abstract

Translocated captive-bred predators are less skilled at hunting than wild-born predators and more prone to starvation post-release. Foraging in an unfamiliar environment presents many further risks to translocated animals. Knowledge of the diet and foraging behaviour of translocated animals is therefore an important consideration of reintroductions. We investigated the diet of the endangered meso-predator, the eastern quoll *Dasyurus viverrinus*. We also opportunistically observed foraging behaviour, enabling us to examine risks associated with foraging. Sixty captive-bred eastern quolls were reintroduced to an unfenced reserve on mainland Australia (where introduced predators are managed) over a two year period (2018, 2019). Quolls were supplementary fed macropod meat but were also able to forage freely. Dietary analysis of scats (n = 56) revealed that quolls ate macropods, small mammals, birds, invertebrates, fish, reptiles and frogs, with some between-year differences in the frequency of different diet categories. We also observed quolls hunting live prey. Quolls utilised supplementary feeding stations, indicating that this may be an important strategy during the establishment phase. Our study demonstrated that, in a novel environment, captive-bred quolls were able to locate food and hunt live prey. However, foraging was not without risks; with the ingestion of toxic substances and foraging in dangerous environments found to be potentially harmful. Knowledge of the diet of reintroduced fauna in natural landscapes is important for understanding foraging behaviour and evaluating habitat suitability for future translocations and management.

## Introduction

Translocated animals need to recognise and locate food to survive in their new environment [1,2]. Inefficient foraging is energy intensive, contributing to exhaustion, malnutrition, and starvation [1,3,4]. Loss of body condition, particularly during energetically demanding or stressful periods, can contribute to increased susceptibility to disease [5] and has a cost to fitness [e.g. decreased reproduction, 6]. Inefficient foraging through lack of experience can result in mortality from ingesting harmful food resources [7] or lead to increased exposure to predators [1,8]. In the initial period following release, translocated animals are especially vulnerable to mortality as they adapt to changes in foraging conditions and adjust to novel threats in an unfamiliar environment [7].

Foraging behaviour in wild environments is dependent on the availability, density and distribution of resources [9]. For translocated fauna, identifying suitable resources can be informed by past experiences, with familiar resources being more readily recognised [10]. Interactions with conspecifics can further inform food availability [11], with foraging cues gleaned from copying more experienced individuals [2]. Conversely, competition for resources may lead to sub-optimal foraging [12]. Intraspecific dominance can drive subordinates out of productive areas [13,14], and interspecific competition may restrict foraging, both temporally and spatially [15,16]. Translocated animals need to adapt quickly to new foraging conditions, lest they starve. Captive-bred animals are particularly vulnerable to starvation and loss of body condition [17], particularly those reliant on specialised foraging [1,4,18] and or hunting skills [3].

We report on the diet and foraging behaviour of a reintroduced captive-bred marsupial predator, the eastern quoll *Dasyurus viverrinus*. The species was first translocated to the wild on mainland Australia in 2018, following their decline and presumed extinction last century [19]. The long absence of the eastern quoll on the Australian mainland limits our knowledge of their foraging behaviour and requirements in the wild. Studies in Tasmania, where the species is still extant, reveals a diverse diet that includes invertebrates, small vertebrates and carrion [15,20–22]. The species scavenges and hunts independently [22], and is an opportunistic forager, adapting to novel food resources [e.g. introduced rabbits *Oryctolagus cuniculus* and chickens *Gallus gallus domesticus*, 23], and seasonally available prey that is related to yearly weather fluctuations [21].

In an earlier related study, Robinson et al. [24] conducted an *a priori* risk assessment for the translocation to the mainland of this captive-bred marsupial predator. That study identified several threats including loss of body condition, ingestion of harmful substances (e.g. poison baits) and predation by introduced predators (e.g. the red fox *Vulpes vulpes*) and native predators (e.g. Diamond python *Morelia spilota*). Foxes were subsequently confirmed to be a key threat limiting the successful re-establishment of the eastern quoll on the mainland [24]. Poison baits containing 1080 are used to control the red fox and are an effective measure for reducing their numbers [25] but there are concerns about bait take by non-target animals. The *a priori* risk assessment [24] considered the risk of poisoning to be low for the eastern quoll due to their relatively higher tolerance to the active compound (sodium fluroacetate) in the bait [26]. Baker et al. [27] also predicted that predation by eastern quolls may adversely affect endangered species at the translocation site, including the eastern bristlebird *Dasyornis brachypterus*, and a recently translocated population of southern brown bandicoots *Isoodon obesulus obesulus* [28]. Robinson et al. [24] considered the risk of predation by quolls on these species as minor due to differences in preferred habitat between quolls [typically open grassland, farmland and forest ecotones, 29] and these endangered prey species [e.g. typically low and dense vegetation such as heath, 28,30].

The key questions we posed in this study were: 1) What is the dietary composition of captive-bred eastern quolls translocated to mainland Australia, and does it vary between years?; 2) Is there evidence of wild foraging and or hunting?; and 3) Does foraging by the eastern quoll pose risks to itself or other endangered species, as predicted by earlier studies [24,27]? We predicted that the diet of translocated eastern quolls would be diverse, reflecting the species’ foraging behaviour [21], but similar between years due to comparable timing of release and comparable climate statistics for the two years [31]. We further predicted that quolls would forage beyond supplementary feed stations but that risks associated with foraging would be minimal due to mitigation strategies outlined in [24].

## Materials and methods

### Study species

**Fig 1:**
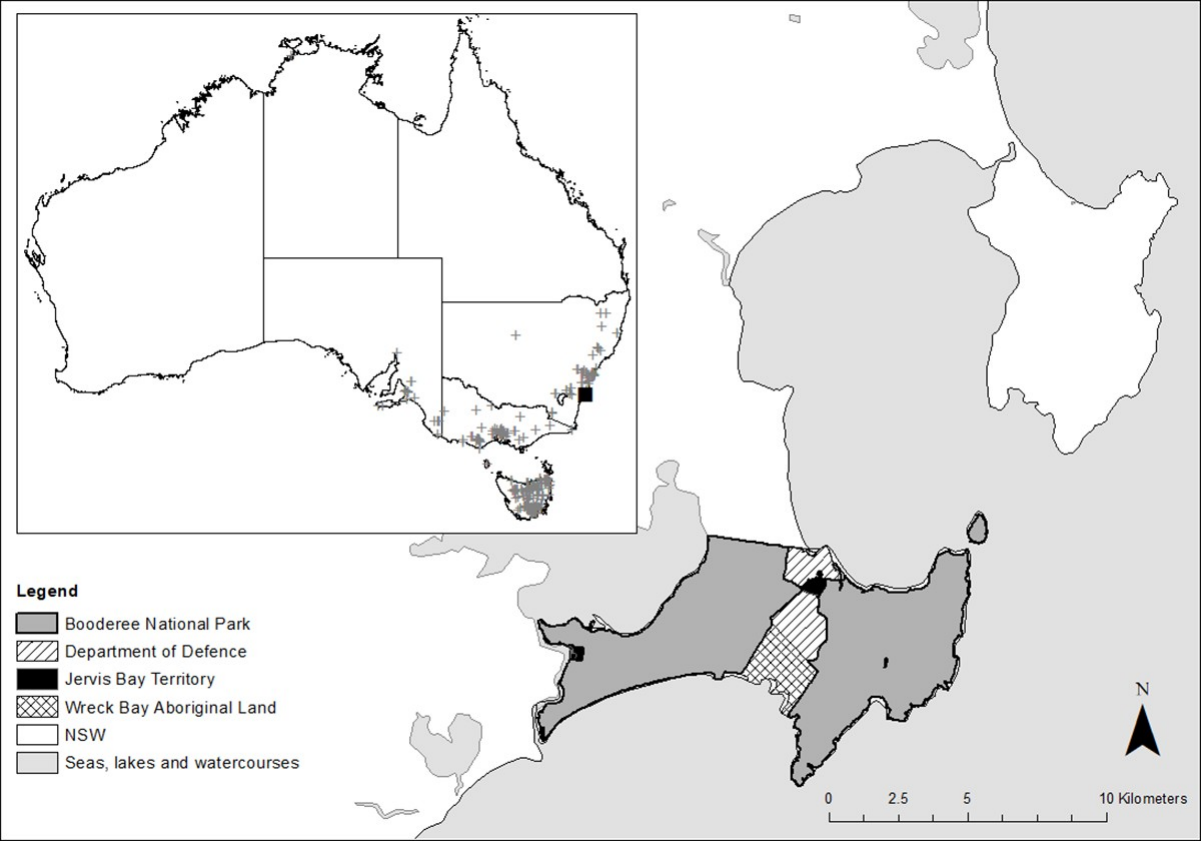
Historic distribution of the eastern quoll in Australia [grey crosses, 36] and the release point for the species at Booderee National Park (black square).

The eastern quoll is a sexually dimorphic marsupial predator. Adult males average 1250 g (range 900–2000 g) compared with smaller females that average 850 g (700–1100 g). The species was historically common across south-eastern Australia (Fig 1), occurring in a variety of habitats such as grassland, farmland, forest and coastal areas [32]. The species declined due to disease and predation by introduced carnivores [33,34]; it is currently listed as endangered under the *Environment Protection and Biodiversity Conservation (EPBC) Act 1999* and the IUCN Red List of Threatened Species [35].

### Reintroduction to mainland Australia

We reintroduced 60 captive-bred eastern quolls to an unfenced, introduced predator-managed reserve on mainland Australia over two consecutive years (March 2018, April/May 2019). The release location was Booderee National Park (BNP), a 6,400ha coastal reserve located in south-eastern Australia (Fig 1). The reserve is co-managed between Traditional Owners (the Wreck Bay people), and Parks Australia [37]. Since 1999, managers at BNP have maintained an introduced predator control program using FOXOFF® 1080 manufactured poison baits, for the control of the introduced red fox [25].

### Feeding regime before and after translocation

Translocated eastern quolls were raised in captivity at three sanctuaries (Trowunna Wildlife Sanctuary, Devils@Cradle and Aussie Ark). The feeding regime was similar between all sanctuaries, with quolls typically fed six days per week, with one day of fasting. Feeding consisted of a diet supplement mix (e.g. carrot, apple, egg, sardine, manufactured carnivore mix), alternated with marsupial (mainly macropod) or chicken carcass pieces. Live prey was not a component of the feeding program, however, small prey (e.g. insects, small lizards) were able to enter the enclosures.

To minimise loss of body condition and assist with the transition to wild foraging, we provided supplementary feeding stations within the release environment. Initially, supplementary food consisted of a prepared quoll mix (macropod mince, grated carrot, grated apple) similar to the sanctuary feeding regime, and then later transitioned to macropod carcass and diced macropod (~ 500 g). We maintained a maximum of six feeding stations at one time and stations were re-stocked twice a week. If we did not detect a quoll using a particular station, we closed that station.

### Monitoring foraging and movements

We installed up to two camera traps at every feeding station (2018 = 10 cameras, 2019 = 11 cameras) to monitor the use by the eastern quoll. We set up cameras to face the feeding station, approximately 2–5 metres away. We cleared vegetation in the field of view to maximise the chance of capturing clear photos of animals. We set the cameras to capture motion triggered still photos. We recorded quoll foraging behaviour captured on cameras along with incidental observations during routine monitoring [24] and/or reported by residents and visitors to the park. Observations were verified where possible by predation remains, photographic evidence, and or reliable observer.

We used VHF/GPS collars to monitor the movements of 41 quolls. We fit collars to 20 animals in 2018 (Telemetry Solutions, model FLR V LS14250, Concord, CA) and 21 animals in 2019 (Sirtrack, model ZV6C 163 Zilco VHF Collar). Quolls were tracked daily for the first four weeks following release, then at least three times per week until day 45. After this period, quolls were tracked less regularly (approximately once per week) until collars were removed following breeding (up to 100 days). Locations and trajectories of quoll movements were visually assessed and compared with mapped vegetation layers [38] in ArcMap 10.8.1. Our research was conducted in strict accordance with the recommendations in the Australian Code for the care and use of animals for scientific purposes [39]. The protocols were approved by The Australian National University Animal Experimentation Ethics committee (Protocol Numbers: A2016/30 and A2018/71).

### Scat collection and analysis

We collected scats opportunistically from traps containing quolls (70% of all samples) and from areas known to be occupied by quolls from our tracking. Quoll scats could not be confused with scats from other species as they have a characteristic shape and smell [40], and no other quoll species has been recorded at BNP within the last 15 years of fauna monitoring [41]. We stored scats in well ventilated, dry paper bags before they were analysed and verified as quoll scats by a specialist (G. Story, Scatsabout, Majors Creek, NSW). Processing and analysing scats involved separating out each dietary item, identifying it to the lowest possible taxonomic group, and visually estimating the percent volume for each component of each scat. We assigned each dietary item to one of nine categories: macropod (or supplementary feed), eastern quoll, other mammal, bird, herpetofauna, fish, invertebrate, vegetation, or other (e.g. non organic). Our categories were not the same as those in other studies of the species diet [e.g. 21,22]. We included additional categories (e.g. herpetofauna), and grouped together lower taxonomic classes and or life stage classes of invertebrates, as not all categories could not be identified to species level.

We analysed compositional differences in scats based on two measures, the frequency of occurrence (FO) and percent volume (PV) according to diet categories described above. The frequency of occurrence is the most commonly reported measure for carnivore dietary studies [42], but it tends to overestimate the importance of smaller food items. Percent volume provides a quantitative relative measure, however, it may underestimate easily digestible dietary items such as soft-bodied animals. Both metrics are recommended to assess the relative importance of food items in carnivore diets [42].

To compare the rates of frequency of occurrence of each of the potential diet items between 2018 and 2019, we employed Bayesian logistic regression [43]. We used the brms package [44] in R 3.6.3 [45]. We used student t priors with 7 degrees of freedom with location 0 and scale 2.5 for the model parameters to avoid problems with complete separation in logistic regression [46]. For each model, we ran 2000 iterations of the Markov Chain and discarded the first 1000 as a warmup, Gelman and Rubin’s Rhat statistic was used to assess convergence [47] and it was adequate in all cases with Rhat < 1.01. We report log odds ratios for 2019 relative to 2018 with 95% credible intervals. Credible intervals that don’t overlap zero indicate evidence of a difference between the years. Log odds ratios that are greater than zero indicate that the diet item was more likely in 2019 compared to 2018 and log odds ratios less than zero indicate the diet item was more common in 2018 compared to 2019.

## Results

### Scat analysis

**Fig 2:**
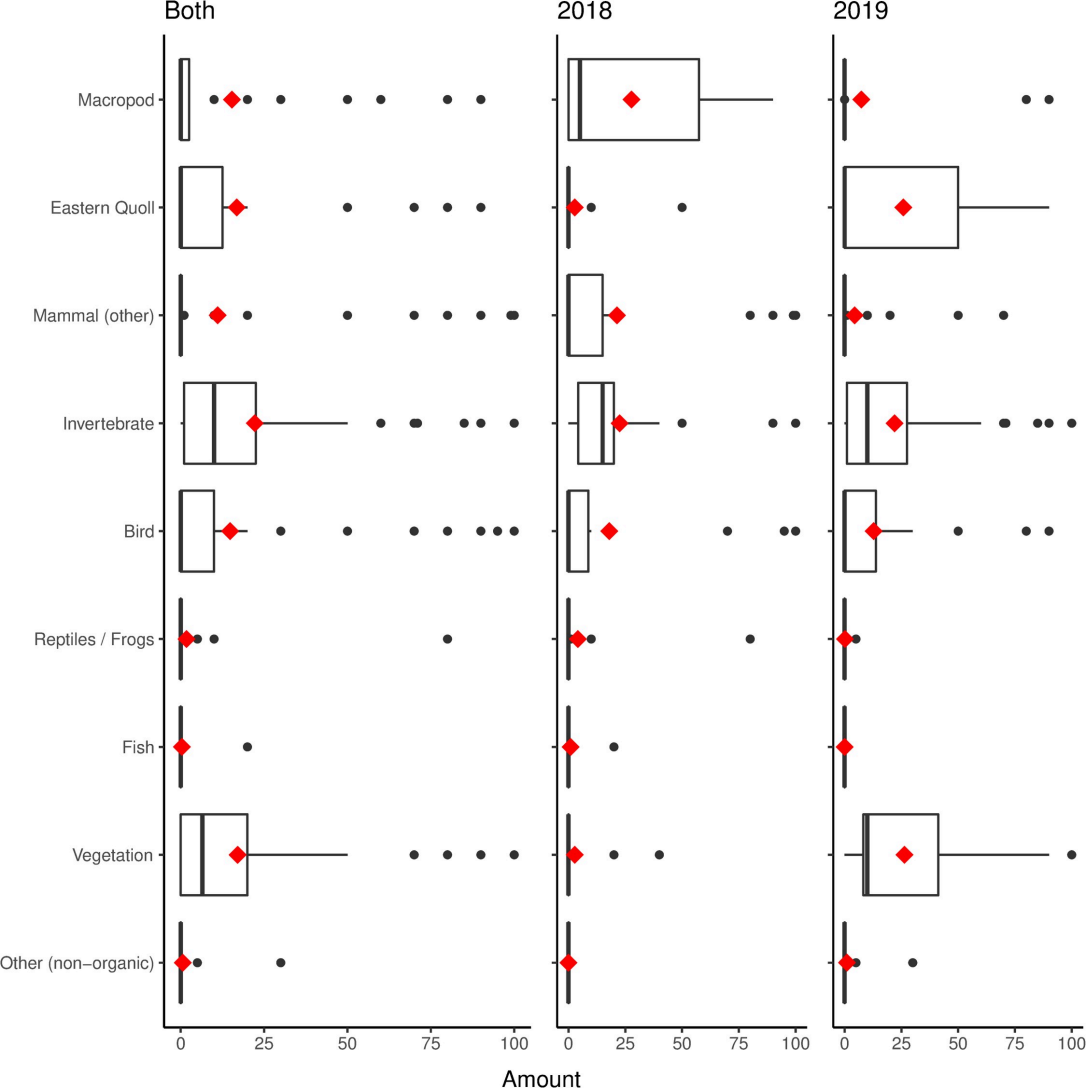
Boxplot of proportional volumes (PV) per diet category for both years combined (both) and individually for 2018 (n = 22) and 2019 (n = 34). The red diamond represents the mean and the heavy line represents the median.

Our scat analysis revealed that translocated eastern quolls ate macropods, small mammals, invertebrates, birds, herpetofauna, fish, vegetation and other non-organic material (Table 1, Fig 2). Across both years, at least 10% of the proportional volume (PV) of scats contained invertebrates, vegetation, macropods, other mammals and birds; these same items were also most frequent (≥ 20% FO, Table 1). We often found the remains of eastern quoll in scats (> 50% FO, Table 1). However, more than half of these occurrences were due to traces of eastern quoll hair (22.4% FO with < 0.01 PV removed, Table 1). There were instances of higher volumes of eastern quoll remains in scats, with some scats containing up to 90% by proportional volume (PV) per scat (Fig 2), and one scat containing bone fragments.

**Table 1:** Average proportional volume (PV) and frequency of occurrence (FO) for all diet categories found in eastern quoll scats (n = 56).

Category	Average proportional volume (PV)	Frequency of Occurrence (FO)	FO (with eastern quoll records of 0.01 removed)
Macropod	15.4	26.8	25.0
Eastern quoll	16.8	62.5	26.8
Mammal (other)	11.1	19.6	19.6
Invertebrate	22.2	80.4	80.4
Bird	14.8	35.7	35.7
Reptiles/Frogs	1.7	8.9	8.9
Fish	0.4	1.8	1.8
Vegetation	17.1	53.6	53.6
Other (non-organic)	0.6	3.6	3.6

**Fig 3:**
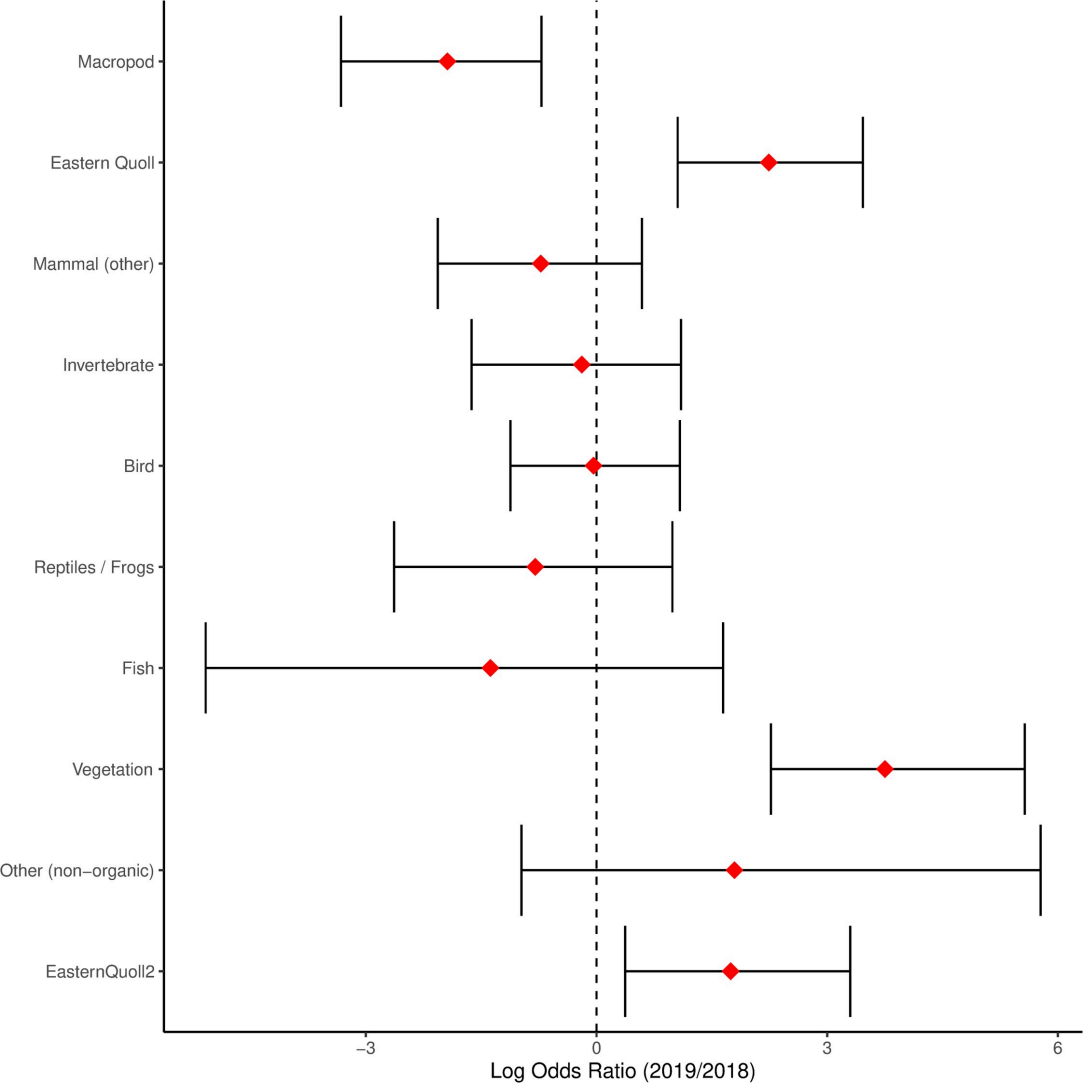
Log Odds Ratio comparing the frequency of occurrence (FO) of diet categories between years. Bars (95% credible intervals) not overlapping zero indicate a difference between years. Eastern quoll is presented with all records (eastern quoll) and with records of 0.01 removed (eastern quoll 2).

There were some differences in the frequency of occurrence (FO) of diet categories between years. The results of the Bayesian logistic regression (Fig 3) revealed that macropods were more common in the scats of quolls in 2018 compared to 2019 (Log odds ratio (LOR) = -1.93, 95% credible interval CI [-3.32, -0.72]). In contrast, vegetation was more common in 2019 compared to 2018 (LOR = 3.75, 95% CI [2.27, 5.56]) and the presence of quoll was more common in 2019 compared to 2018 (LOR = 2.24, 95% CI [1.06, 3.45] for all samples; LOR = 1.74, 95% CI [0.37, 3.30] excluding trace amounts of quoll). Non-organic material (plastic) was detected only in 2019 (Figs 2 and 3); FO and PV for both years are provided in S1. A full list of identified diet components is provided in Table 2, and the percent volume per scat in S2.

**Table 2:** Summary of diet components identified in each category.

Diet category	Species (or lowest possible taxonomic class)
Macropod	*Macropus giganteus*
	*Wallabia bicolor*
Eastern quoll	*Dasyurus viverrinus*
Mammal (other)	*Oryctolagus cuniculus*
	*Perameles nasuta*
	*Rattus fuscipes*
	*Trichosurus vulpecula*
	Mammal (other)
Invertebrate	Ant
	Beetle
	Centipede
	Cocoon
	Crustacean
	Snail
Bird	*Eudyptula minor*
	Bird (other)
Herpetofauna	Dragon
	Frog
	Skink
	Snake
Fish	Fish
Vegetation	Vegetation
Other (non-organic)	Non-organic material (plastic)

### Observations and movements

**Fig 4:**
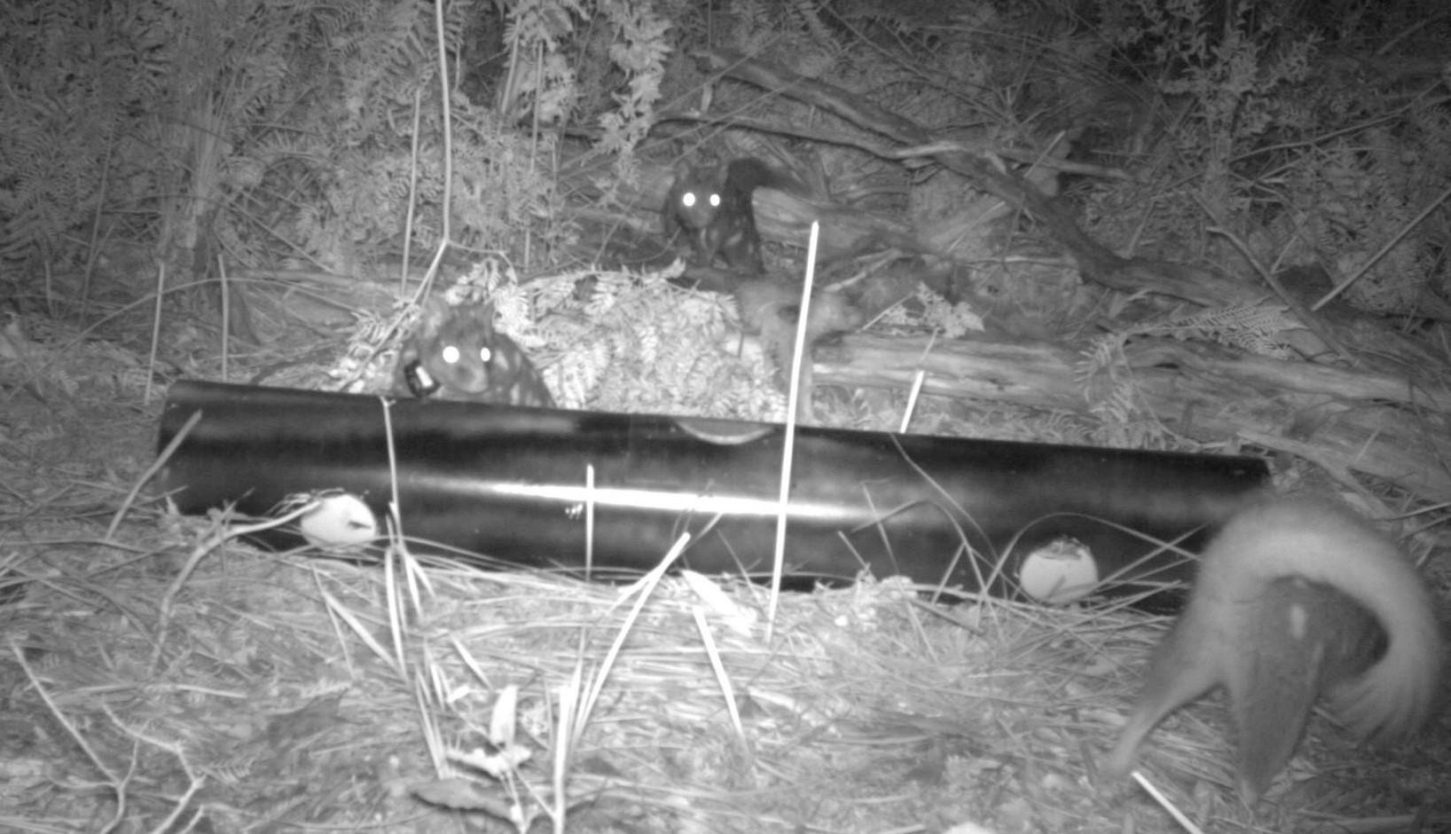
Eastern quolls feeding at a supplementary feed station within Booderee National Park. Photo credit Parks Australia.

**Fig 5:**
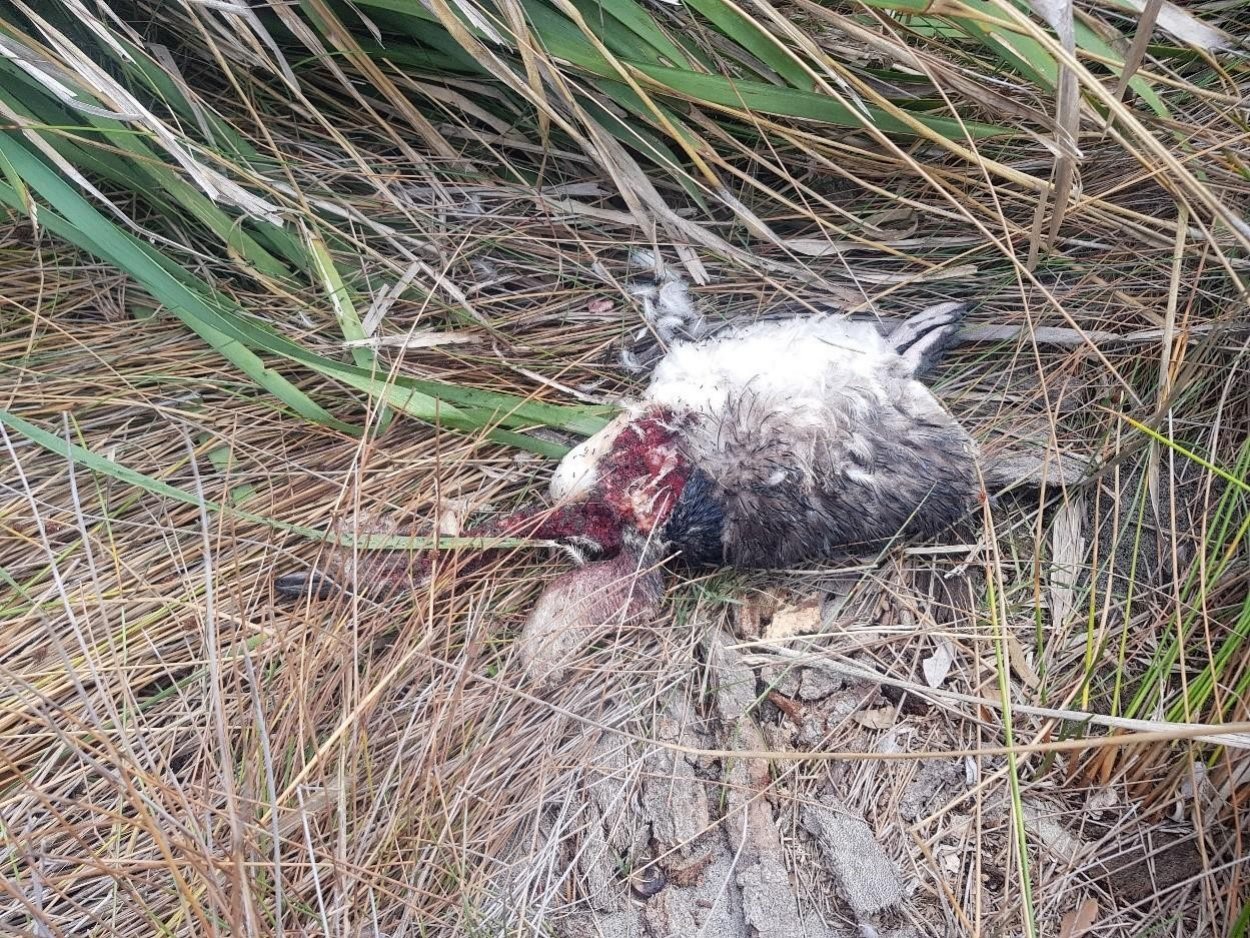
Remains of a little penguin found predated by eastern quoll at St Georges Head, Booderee National Park. Photo credit D. Maple, Parks Australia.

We observed the eastern quoll on camera feeding at supplementary feed stations, either individually or in groups of up to three animals (Fig 4). We directly observed the eastern quoll catching and feeding on moths (*Abantiades hyalinatus*), however, the remains of moths were not detected in scats (Table 1). We found the remains of a little penguin *Eudyptula minor*, with evidence of predation (or scavenging) within 1 m of a known quoll den (Fig 5); predation by quoll was assumed due to the bite marks being consistent with quoll-sized predator and a lack of caching that would indicate fox. We later confirmed the remains of a penguin within a quoll scat (Table 2). Local residents reported adult quolls taking food scraps, predating on domestic chickens, and juvenile quolls entering pet enclosures, presumably attracted to pet food [24]. We detected quolls in a variety of vegetation communities, including heath. Visual assessment of movement trajectories indicated that the animals were transient within heath, with subsequent locations recorded in other habitat types (e.g. forest).

## Discussion

We present preliminary findings of the diet of a population of reintroduced captive-bred native predators on mainland Australia. We found that reintroduced eastern quolls consumed a diverse range of food, including live prey. Their ability to hunt in the wild is an important finding considering their limited exposure to hunting in captivity. Based on opportunistic foraging observations and scat contents, we discuss potential negative impacts of predation by the eastern quoll on existing species at BNP, and the risk of the eastern quoll consuming harmful or toxic resources.

### Diet of a reintroduced native predator

Similar to past studies of the eastern quoll [15,20–22], we found that reintroduced captive-bred quolls consumed a variety of prey, carrion and vegetation. Likewise, a study of the Tasmanian devil *Sarcophilus harrisii* found that the diet of translocated captive-bred devils was similar to wild devils; there were differences in proportions of food items but this possibly reflected resource availability [48].

We commonly found remains of the eastern quoll in scats of translocated quolls. However, this was largely traces of hair and was less than 0.01 by proportional volume (PV). Traces of quoll hair likely reflects self-grooming or biting other quolls which is common during breeding [22]; a period which overlapped with scat collection, especially in 2019 (due to the timing of the release). We found one scat containing bone fragments of an adult quoll, suggesting cannibalism either by predation or scavenging; reports of cannibalism in the eastern quoll have been previously noted [21,22]. Vegetation was found in high quantities in some scats; this could be due to incidental ingestion when preying on invertebrates [20,21]. Plastic was detected in two scats in 2019, with one sample being identified as soft plastic fishing lure. The eastern quoll fed regularly at supplementary feeding stations and macropods were found in scats, indicating that the feeding stations were likely an important strategy for minimising loss in body condition [24].

### Foraging and hunting ability

We found evidence that quolls captured small prey (e.g. invertebrates) and larger prey (e.g. birds) indicating that, despite being captive-bred, translocated quolls were able to hunt successfully. Earlier work has demonstrated that captive-bred eastern quolls, with no prior hunting experience, are able to kill live prey [49], suggesting the species has innate hunting abilities. However, that experiment occurred in captivity, with prey not able to easily escape, and not every attempted kill was successful [49]. Efficiency and success of hunting by captive-bred predators improves with learning and experience [50].

Diet flexibility in the eastern quoll may have facilitated an easier transition from captive to wild conditions than other captive-bred animals reliant on specific food resources and/or which have low rates of energy intake [e.g. giant panda *Ailuropoda melanleuca*, 18]. The eastern quoll has successfully adapted to novel prey [e.g. rabbits, 23], and, in Tasmania (where wild populations of the species remain), the diet reflects seasonally available resources [21].

### Risks of foraging to the eastern quoll and other species

The ability of the eastern quoll to hunt other native animals was previously identified as a risk to populations of threatened species at BNP [27]. However, our monitoring-to-date indicates that the eastern quoll is not threatening the persistence of any endangered species at BNP; there is no evidence in quoll scats or from observations of their foraging and movements that the eastern quoll has predated on the eastern bristlebird or on the recently reintroduced southern brown bandicoot. This contrasts with the reintroduction of the western quoll *Dasyurus geoffroii* into a large fenced reserve, where the remains of four threatened species were found in scats [51]. Despite this, predation by the western quoll was not found to cause a decline in the abundance of threatened species; likely due to relatively high numbers of threatened prey species in the enclosure compared with numbers of reintroduced quolls [51]. Populations of threatened species still persist at BNP, and there is no evidence of recent decline based on the reintroduction of quolls (pers. comm N.Dexter). We did, however, find evidence that translocated captive-bred quolls are capable of hunting similar sized prey (e.g. little penguin, adult mass 1.5 kg). Little penguins breed on offshore islands and occasionally come ashore at BNP. They are listed as least concern under the *EPBC Act 1999* and predation by the eastern quoll does not currently present a threat to their persistence. Our understanding of quoll foraging behaviour at BNP is based on sporadic and opportunistic observations. It is important to continue to monitor the impact of predation by the eastern quoll, especially if circumstances change (e.g. increase in quoll population size, change in threat status of prey items).

The risk that reintroduced eastern quolls could forage on toxic substances (e.g. 1080 fox baits) was *a priori* identified, and confirmed as a low risk [24]. It is possible that the eastern quoll could ingest other harmful substances (e.g. rodenticides) either directly or via secondary poisoning [52]. Predation of novel prey can also be harmful to quolls, either via poisoning [e.g. consumption of the cane toad *Rhinella marina* by the northern quoll *Dasyurus hallucatus*, 7], or by injury. Foraging also could prove dangerous to quolls when conducted in risky environments. This includes areas attractive to predators of quolls, along roadsides with potential for collisions with vehicles, and within human-occupied areas where there are dogs [24]. However, we are limited in this study to examine all these foraging risks. We conducted post mortems and collected livers of deceased quolls to identify cause of death. Low concentrations of anticoagulant rodenticides (brodifacoum, difenacoum) were found (M.Lohr pers.comm.) but deaths were attributed to other factors, and not poisoning [24]. To reduce risks associated with foraging of the eastern quoll, the staff at BNP employ strategies including introduced predator control, speed restrictions of vehicles, and wildlife warning signs for road users. We further advocate responsible pet ownership of adjacent properties by encouraging pet containment and installation of quoll-proof barriers on outdoor dog runs, and limiting the amount of pet food dispensed to minimise attraction of the eastern quoll.

## Conclusion

Our study provides an initial assessment of the diet and foraging behaviour of the reintroduced captive-bred eastern quoll to the wild on mainland Australia. Studies such as ours are important for revealing early findings on the adequacy of resources in the release environment and the responses by translocated animals. We have evidence to suggest that the eastern quoll is able to adjust to novel food resources and hunt for live prey. However, supplementary feeding is likely to be essential for assisting with their transition from captive to wild. We recommend that managers consider risks and the mitigation of those risks prior to the translocation of captive-bred predators; this includes risks to the translocated animal in terms of food availability, foraging skills, and susceptibility to declines in body condition, as well as impacts to prey species in the release environment.

Our study was limited in being able to examine definitively the foraging behaviour of translocated quolls and the risks to themselves or prey species in the release environment. Future studies could investigate quoll movements in relation to prey species, predator/prey abundance through time, shifts in the use of habitat by prey, and diet in relation to seasonal prey availability and body condition. Ongoing monitoring of foraging habits and diet remains important to ensure appropriate management of risks to the eastern quoll and other species, and can provide important insights for translocations of other native captive-bred predators into wild environments.

## Supporting information

S1 Fig (TIF)

S1 Table Proportional volume (PV) and Frequency of Occurrence (FO) for all diet categories found in eastern quoll scats according to the year of collection (2018 = 22, 2019 = 34). (DOCX)

S2 Table Percent volume (visual estimate) of diet categories found in *Dasyurus viverrinus* scats in 2018 (n = 22) and 2019 (n = 34). (DOCX)
