# Supplementary figures and images for: Finding food in a novel environment: The diet of a reintroduced endangered meso-predator to mainland Australia, with notes on foraging behaviour
Source: PLoS One. 2020 Dec 17;15(12):e0243937. doi: 10.1371/journal.pone.0243937 (PMC7746155; doi:10.1371/journal.pone.0243937)

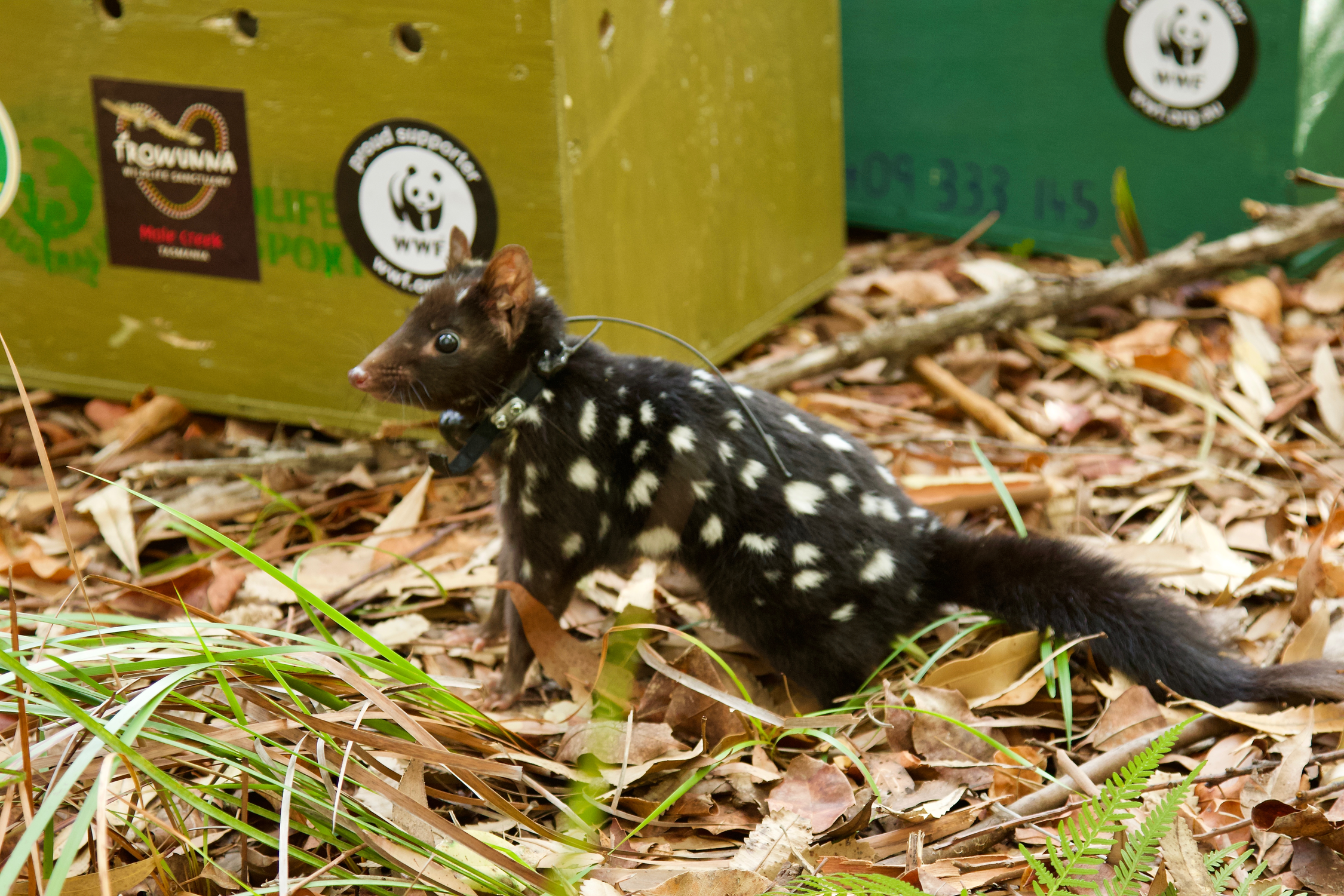

Supplement: S1 Fig — (TIF) [file pone.0243937.s001.tif]
