# Supplementary material for: Finding food in a novel environment: The diet of a reintroduced endangered meso-predator to mainland Australia, with notes on foraging behaviour
Source: PLoS One. 2020 Dec 17;15(12):e0243937. doi: 10.1371/journal.pone.0243937 (PMC7746155; doi:10.1371/journal.pone.0243937)
Supplement: S1 Table — (DOCX) [file pone.0243937.s002.docx]

S1. Proportional volume (PV) and Frequency of Occurrence (FO) for all diet categories found in eastern quoll scats according to the year of collection (2018 = 22, 2019 = 34)

|  | Average proportional volume (PV) | | Frequency of Occurrence (FO) | | Frequency of Occurrence (with eastern quoll records of 0.01 removed) | |
| --- | --- | --- | --- | --- | --- | --- |
| Category | 2018 | 2019 | 2018 | 2019 | 2018 | 2019 |
| Macropod | 27.7 | 7.4 | 50.0 | 11.8 | 50.0 | 8.8 |
| Eastern Quoll | 2.7 | 25.9 | 31.8 | 82.4 | 9.1 | 38.2 |
| Mammal (other) | 21.3 | 4.4 | 27.3 | 14.7 | 27.3 | 14.7 |
| Invertebrate | 22.5 | 22.0 | 81.8 | 79.4 | 81.8 | 79.4 |
| Bird | 18.0 | 12.8 | 36.4 | 35.3 | 36.4 | 35.3 |
| Reptiles / Frogs | 4.1 | 0.2 | 13.6 | 5.9 | 13.6 | 5.9 |
| Fish | 0.9 | 0.0 | 4.5 | 0.0 | 4.5 | 0.0 |
| Vegetation | 2.7 | 26.3 | 9.1 | 82.4 | 9.1 | 82.4 |
| Other (non-organic) | 0.0 | 1.0 | 0.0 | 5.9 | 0.0 | 5.9 |
