# Supplementary material for: Finding food in a novel environment: The diet of a reintroduced endangered meso-predator to mainland Australia, with notes on foraging behaviour
Source: PLoS One. 2020 Dec 17;15(12):e0243937. doi: 10.1371/journal.pone.0243937 (PMC7746155; doi:10.1371/journal.pone.0243937)
Supplement: S2 Table — (DOCX) [file pone.0243937.s003.docx]

S2. Percent volume (visual estimate) of diet categories found in *Dasyurus viverrinus* scats in 2018 (n = 22) and 2019 (n = 34).

|  |  | Macropod | | Eastern quoll | Mammal (other) | | | | | Invertebrate | | | | | | Bird | | Herpetofauna | | | | Fish | Vegetation | Other |
| --- | --- | --- | --- | --- | --- | --- | --- | --- | --- | --- | --- | --- | --- | --- | --- | --- | --- | --- | --- | --- | --- | --- | --- | --- |
| No | Year | *Wallabia bicolor* | *Macropus giganteus* | *Dasyurus viverrinus* | *Trichosurus vulpecula* | *Rattus fuscipes* | *Perameles nasuta* | *Oryctolagus cuniculus* | Mammal (other) | Beetle | Snail | Centipede | Crustacean | Ant | Cocoon | Bird | Penguin | Snake | Skink | Dragon | Frog | Fish | Vegetation | Non organic material |
| 1 | 2018 |  | 50 |  |  |  |  |  |  | 50 |  |  |  |  |  |  |  |  |  |  |  |  |  |  |
| 2 | 2018 |  | 80 |  |  |  |  |  |  | 20 |  |  |  |  |  |  |  |  |  |  |  |  |  |  |
| 3 | 2018 | 80 |  |  |  |  |  |  |  | 20 |  |  |  |  |  |  |  |  |  |  |  |  |  |  |
| 4 | 2018 |  |  |  |  | 99 |  |  |  | 1 |  |  |  |  |  |  |  |  |  |  |  |  |  |  |
| 5 | 2018 |  |  |  |  |  |  |  |  |  |  |  |  |  |  | 100 |  |  |  |  |  |  |  |  |
| 6 | 2018 |  |  |  |  |  |  |  |  | 5 |  |  |  |  |  | 95 |  |  |  |  |  |  |  |  |
| 7 | 2018 |  |  | 0.01 |  |  |  |  |  |  |  |  |  |  |  | 100 |  |  |  |  |  |  |  |  |
| 8 | 2018 |  |  | 0.01 | 80 |  |  |  |  | 10 | 10 |  |  |  |  |  |  |  |  |  |  |  |  |  |
| 9 | 2018 | 30 |  |  |  |  |  |  |  | 20 |  |  |  |  |  |  |  |  | 10 |  |  |  | 40 |  |
| 10 | 2018 | 50 |  |  |  |  |  |  |  | 50 |  |  |  |  |  |  |  |  |  |  |  |  |  |  |
| 11 | 2018 |  | 80 |  |  |  |  |  |  | 20 |  |  |  |  |  |  |  |  |  |  |  |  |  |  |
| 12 | 2018 |  |  | 0.01 | 50 |  |  | 30 |  |  |  |  |  |  |  |  |  |  |  |  |  |  | 20 |  |
| 13 | 2018 |  |  |  | 100 |  |  |  |  |  |  |  |  |  |  |  |  |  |  |  |  |  |  |  |
| 14 | 2018 | 10 |  | 0.01 |  |  |  |  |  | 90 |  |  |  |  |  |  |  |  |  |  |  |  |  |  |
| 15 | 2018 |  |  | 50 |  |  |  |  |  | 5 |  | 30 |  | 5 |  | 10 |  |  |  |  |  |  |  |  |
| 16 | 2018 |  |  |  |  |  |  |  |  | 5 |  |  | 95 |  |  |  |  |  |  |  |  |  |  |  |
| 17 | 2018 | 90 |  |  |  |  |  |  |  | 10 |  |  |  |  |  |  |  |  |  |  |  |  |  |  |
| 18 | 2018 | 20 |  |  |  |  |  |  |  | 10 |  |  |  |  |  |  | 70 |  |  |  |  |  |  |  |
| 19 | 2018 |  |  | 10 |  |  |  |  |  | 5 |  |  |  |  |  | 5 |  | 80 |  |  |  |  |  |  |
| 20 | 2018 |  |  |  |  |  | 90 |  |  | 3 |  |  |  | 1 |  | 5 |  |  |  | 1 |  |  |  |  |
| 21 | 2018 | 60 |  |  | 20 |  |  |  |  | 20 |  |  |  |  |  |  |  |  |  |  |  |  |  |  |
| 22 | 2018 | 60 |  | 0.01 |  |  |  |  |  | 10 |  |  |  |  |  | 10 |  |  |  |  |  | 20 |  |  |
| 23 | 2019 |  |  | 70 |  |  |  |  |  | 10 |  |  |  | 1 |  |  |  |  |  |  |  |  | 19 |  |
| 24 | 2019 |  |  | 0.01 |  |  |  | 20 |  | 70 |  |  |  |  |  |  |  |  |  |  |  |  | 10 |  |
| 25 | 2019 | 0.01 |  |  |  |  |  |  |  |  |  |  |  |  |  |  |  |  |  |  |  |  | 100 |  |
| 26 | 2019 |  |  | 0.01 |  |  |  |  |  | 5 |  |  |  |  | 25 |  |  |  |  |  |  |  | 70 |  |
| 27 | 2019 |  |  | 90 |  |  |  |  |  | 1 |  |  |  | 1 |  |  |  |  |  |  |  |  | 8 |  |
| 28 | 2019 |  |  | 80 |  |  |  |  |  | 10 |  |  |  |  |  |  |  |  |  |  |  |  | 10 |  |
| 29 | 2019 |  |  | 50 |  |  |  |  |  |  |  |  |  | 5 |  |  |  |  |  |  |  |  | 45 |  |
| 30 | 2019 |  |  | 90 |  |  |  |  |  |  |  |  |  | 1 |  |  |  |  |  |  |  |  | 9 |  |
| 31 | 2019 |  |  | 90 |  |  |  |  |  | 1 |  |  |  |  |  |  |  |  |  |  |  |  | 9 |  |
| 32 | 2019 |  |  | 50 |  |  |  |  |  |  |  |  |  |  |  |  |  |  |  |  |  |  | 50 |  |
| 33 | 2019 |  |  | 0.01 |  |  |  |  | 10 | 40 |  |  | 10 | 10 |  |  |  |  |  |  |  |  | 30 |  |
| 34 | 2019 |  |  | 0.01 |  |  |  |  |  |  |  |  |  | 1 |  | 80 |  |  |  |  |  |  | 19 |  |
| 35 | 2019 |  |  | 50 |  |  |  |  |  | 20 |  |  |  |  |  |  |  |  |  |  |  |  | 30 |  |
| 36 | 2019 |  |  | 0.01 |  |  |  |  |  | 50 |  |  |  |  |  |  |  |  |  |  |  |  | 50 |  |
| 37 | 2019 |  |  | 0.01 |  |  |  |  |  |  |  |  |  |  |  | 50 |  |  |  |  |  |  | 20 | 30 |
| 38 | 2019 |  |  | 0.01 |  |  | 50 |  |  |  |  |  |  |  |  |  |  |  |  |  |  |  | 50 |  |
| 39 | 2019 |  |  | 0.01 |  |  |  |  |  |  |  |  |  |  |  |  |  |  |  |  |  |  | 100 |  |
| 40 | 2019 |  | 80 | 0.01 |  |  |  |  |  | 10 |  |  |  |  |  |  |  |  |  |  |  |  | 10 |  |
| 41 | 2019 |  |  | 20 |  |  |  |  |  | 50 |  |  |  | 20 | 1 |  |  |  |  |  |  |  | 4 | 5 |
| 42 | 2019 |  |  |  |  |  |  |  |  | 60 |  | 25 |  |  |  |  |  |  |  | 5 |  |  | 10 |  |
| 43 | 2019 | 90 |  | 0.01 |  |  |  |  |  |  |  |  |  | 1 |  |  |  |  |  |  |  |  | 9 |  |
| 44 | 2019 |  |  | 0.01 |  |  |  |  |  |  |  |  |  |  |  | 20 |  |  |  |  |  |  | 80 |  |
| 45 | 2019 |  |  | 0.01 |  |  |  |  |  | 9 |  |  |  | 1 |  | 90 |  |  |  |  |  |  |  |  |
| 46 | 2019 |  |  | 70 |  |  |  |  |  | 1 |  |  |  | 9 |  | 20 |  |  |  |  |  |  |  |  |
| 47 | 2019 |  |  |  |  |  |  |  |  |  |  |  |  |  |  | 10 |  |  |  |  |  |  | 90 |  |
| 48 | 2019 |  |  | 0.01 |  |  | 1 |  |  | 20 |  | 70 |  |  |  |  |  |  |  |  |  |  | 9 |  |
| 49 | 2019 |  |  | 0.01 |  |  |  |  |  | 50 |  |  |  |  |  | 20 |  |  |  |  |  |  | 30 |  |
| 50 | 2019 |  |  | 50 |  |  |  |  |  | 10 |  |  |  |  |  | 30 |  |  |  |  |  |  | 10 |  |
| 51 | 2019 |  |  | 0.01 |  |  | 70 |  |  | 15 |  |  |  |  |  | 15 |  |  |  |  |  |  |  |  |
| 52 | 2019 |  |  |  |  |  |  |  |  |  |  | 95 |  | 5 |  |  |  |  |  |  |  |  |  |  |
| 53 | 2019 |  | 80 |  |  |  |  |  |  |  |  |  |  | 1 |  | 10 |  |  |  |  |  |  | 9 |  |
| 54 | 2019 |  |  | 80 |  |  |  |  |  | 5 |  |  |  |  |  | 10 |  |  |  |  |  |  | 5 |  |
| 55 | 2019 |  |  | 90 |  |  |  |  |  | 9 |  |  |  |  |  |  |  |  |  |  | 1 |  |  |  |
| 56 | 2019 |  |  |  |  |  |  |  |  | 10 |  |  |  | 10 |  | 80 |  |  |  |  |  |  |  |  |
